# Hematologic adverse events following palbociclib dose reduction in patients with hormone receptor–positive/human epidermal growth factor receptor 2–negative advanced breast cancer: pooled analysis from randomized phase 2 and 3 studies

**DOI:** 10.1186/s13058-020-01263-0

**Published:** 2020-03-12

**Authors:** Johannes Ettl, Seock-Ah Im, Jungsil Ro, Norikazu Masuda, Marco Colleoni, Patrick Schnell, Eustratios Bananis, Dongrui R. Lu, Massimo Cristofanilli, Hope S. Rugo, Richard S. Finn

**Affiliations:** 1 Department of Obstetrics and Gynecology, Klinikum rechts der Isar, Technische Universität München, Ismaningerstr. 22, 81675 Munich, Germany; 2 Seoul National University Hospital Cancer Research Institute, Seoul National University College of Medicine, Seoul, South Korea; 3 National Cancer Center, Goyang-si, South Korea; 4 National Hospital Organization Osaka National Hospital, Osaka City, Japan; 5 IEO, European Institute of Oncology, IRCCS, Milan, Italy; 6 Pfizer Oncology, New York, NY USA; 7 Pfizer Inc, La Jolla, CA USA; 8 Robert H. Lurie Cancer Center of Northwestern University, Feinberg School of Medicine, Chicago, IL USA; 9 University of California San Francisco Helen Diller Family Comprehensive Cancer Center, San Francisco, CA USA; 10 David Geffen School of Medicine at University of California Los Angeles, Los Angeles, CA USA

**Keywords:** Palbociclib, Adverse event management, Neutropenia, Dose reductions, Dose modification

## Abstract

**Background:**

Palbociclib improves outcomes for women with hormone receptor–positive/human epidermal growth factor receptor 2–negative advanced breast cancer (HR+/HER2− ABC). Dose reductions are recommended for the management of hematologic toxicities. A previous pooled analysis from the PALOMA clinical trials showed that 36.9% of patients required dose reduction, predominantly during the first 6 months of treatment and with decreasing frequency during subsequent 28-day treatment cycles (C). Previous data have shown that palbociclib dose reductions do not affect efficacy. This pooled, post hoc analysis evaluated the frequency of hematologic adverse events (AEs) before and after palbociclib dose reduction in PALOMA-1, PALOMA-2, and PALOMA-3.

**Methods:**

This analysis evaluated the frequency of hematologic AEs 30 days before dose reduction and during each subsequent treatment from C1 to C6 among patients who required palbociclib dose reduction. Data were pooled from 3 randomized studies. PALOMA-1 was a phase 2, open-label study of postmenopausal patients untreated for ABC receiving palbociclib plus letrozole or letrozole alone. PALOMA-2 was a phase 3, double-blind study of postmenopausal patients untreated for ABC receiving palbociclib plus letrozole or placebo plus letrozole. PALOMA-3 was a phase 3, double-blind study of pre/perimenopausal or postmenopausal patients, whose disease progressed on prior endocrine therapy, receiving palbociclib plus fulvestrant or placebo plus fulvestrant.

**Results:**

A total of 311 (35.5%) patients with HR+/HER2− ABC required a palbociclib dose reduction (93.6% due to AEs) from 125 to 100 mg. Mean patient age was 59.9 years, and 46.9% of patients had visceral disease. Median time to dose reduction was 70 days. The majority of dose reductions occurred within 3 months of starting palbociclib treatment. Incidences of all-grade and grades 3/4 hematologic AEs were lower following dose reduction.

**Conclusions:**

A decrease in frequency and severity of hematologic AEs, including febrile neutropenia, following palbociclib dose reduction was observed, supporting the recommended use of dose reduction in AE management.

**Trial registration:**

These studies were sponsored by Pfizer. ClinicalTrials.gov: NCT00721409; registration date July 24, 2008. ClinicalTrials.gov: NCT01740427; registration date December 4, 2012. ClinicalTrials.gov: NCT01942135; registration date September 13, 2013.

## Introduction

Palbociclib, an oral inhibitor of cyclin-dependent kinase 4/6 (CDK4/6), is indicated in combination with an aromatase inhibitor as first-line treatment for hormone receptor–positive, human epidermal growth factor receptor 2–negative advanced breast cancer (HR+/HER2− ABC) and in combination with fulvestrant in women with HR+/HER2− ABC whose disease progressed on prior endocrine therapy (ET) [1, 2]. Palbociclib extended median progression-free survival (PFS) compared with ET alone in the 3 randomized PALOMA clinical trials (Supplementary Table 1) [3–7]. Moreover, among patients with HR+/HER2− ABC who had sensitivity to previous ET, treatment with palbociclib plus fulvestrant improved overall survival compared with placebo plus fulvestrant (39.7 vs 29.7 months; hazard ratio, 0.72; 95% CI, 0.55–0.94) [8].

In a pooled, long-term safety analysis of PALOMA-1, PALOMA-2, and PALOMA-3, 36.9% of patients receiving palbociclib for treatment of HR+/HER2− ABC required dose reduction. Most dose reductions occurred during the first 6 months of treatment and were less frequent during subsequent 28-day treatment cycles (C) than during the first 6 months [9]. Neutropenia and leukopenia were the most common adverse events (AEs) that led to dose reductions in patients treated with palbociclib [9]. The incidence of hematologic and nonhematologic AEs was highest within the first 2 months of palbociclib treatment, which coincided with the period with the most dose modifications (C1–C3) [9].

Dose reductions and modifications are recommended for the management of hematologic toxicities resulting from palbociclib treatment [1, 2]. Therefore, an analysis of the effect of palbociclib dose modifications on the incidence of hematologic AEs is warranted to evaluate whether neutropenia is adequately managed by dose modifications.

## Methods

Detailed methods for all 3 trials have been previously published [3, 4, 6]; the study designs are shown in Supplementary Figure 1. At the time of data analysis, follow-up was ongoing for PALOMA-2 and PALOMA-3 to collect overall survival data.

### Study design

Data were pooled from 3 randomized trials. PALOMA-1 was a phase 2, open-label study of palbociclib plus letrozole versus letrozole alone in treatment-naive postmenopausal women with estrogen receptor–positive (ER+)/HER2− ABC [3]. Patients were randomized 1:1 to receive palbociclib (125 mg/day, 3 weeks on, 1 week off [3/1 schedule]) plus continuous oral letrozole (2.5 mg/day) or continuous oral letrozole alone. PALOMA-2 was a phase 3, double-blind study of palbociclib plus letrozole versus placebo plus letrozole in treatment-naive postmenopausal women with ER+/HER2− ABC [4, 5]. Patients were randomized 2:1 to receive either palbociclib plus letrozole or placebo plus letrozole using the same doses and schedule as in PALOMA-1. PALOMA-3 was a phase 3, double-blind study of palbociclib plus fulvestrant versus placebo plus fulvestrant in pre/perimenopausal and postmenopausal patients whose disease had progressed on prior ET [6, 7]. Patients were randomized 2:1 to receive palbociclib (125 mg/day, oral, 3/1 schedule) plus fulvestrant (500 mg, intramuscular injection, days 1 and 15 of C1 and on day 1 of every 28-day cycle thereafter) or placebo plus fulvestrant. Pre/perimenopausal women were required to receive a luteinizing hormone-releasing hormone agonist ≥ 4 weeks before study entry and then goserelin throughout the study. Dose reduction and modification guidelines were relatively similar among the component trials (Supplementary Table 2), although PALOMA-3 permitted dosing at 75 mg/day on a 2 weeks on, 2 weeks off schedule if toxicities were not resolved at a 75-mg/day dose on a 3/1 schedule.

### Outcomes and assessments

Hematology assessments were performed on days 1 and 15 for the first 2 cycles and day 1 of each subsequent cycle. The frequency of hematologic AEs was assessed before dose reduction and for the 6 subsequent cycles after dose reduction in patients who had 1 palbociclib dose reduction (125 to 100 mg) and in patients who had a second dose reduction (100 to 75 mg). Time points for AE assessments are shown in Supplementary Table 3. Severity of any-cause AEs was recorded by investigators and graded according to the National Cancer Institute Common Terminology Criteria for Adverse Events v3.0 (PALOMA-1) and v4.0 (PALOMA-2 and PALOMA-3) [3, 4, 6]. For hematologic AEs, related preferred terms were aggregated into the cluster terms neutropenia, leukopenia, thrombocytopenia, and anemia.

### Statistical analyses

Data were analyzed from patients receiving palbociclib who did and did not require a dose reduction in PALOMA-1 (data cutoff: January 2, 2015), PALOMA-2 (data cutoff: February 26, 2016), and PALOMA-3 (data cutoff: July 31, 2015). All data were analyzed descriptively.

## Results

### Patients

The pooled population included a total of 875 patients who received palbociclib plus ET (PALOMA-1, *n* = 84; PALOMA-2, *n* = 444; PALOMA-3, *n* = 347) [3, 4, 6]. Of the pooled population, 311 (35.5%) patients with HR+/HER2− ABC required a palbociclib dose reduction from 125 to 100 mg, and 105 (12%) patients required a second dose reduction from 100 to 75 mg. The majority of dose reductions were due to AEs (93.6%). AEs that led to dose reductions in at least 2% of patients were neutropenia (*n* = 261, 83.9%), leukopenia (*n* = 27, 8.7%), infections (*n* = 11, 3.5%), thrombocytopenia (*n* = 10, 3.2%), fatigue (*n* = 8, 2.6%), and asthenia, febrile neutropenia, and stomatitis (all *n* = 7, 2.3%). Another 3.9% of dose reductions were due to other reasons, and 2.6% had missing reasons. Mean age of patients who received dose reduction was 59.9 years, with the majority of patients < 65 years old (61.7%); two thirds of patients were white (67.8%), nearly half had visceral disease (46.9%), and the majority had ≥ 2 disease sites involved (66.9%; Table 1).

**Table 1:** Demographics and baseline characteristics for patients receiving palbociclib plus endocrine therapy with/without dose reduction

Characteristic	Palbociclib plus endocrine therapy*		
	Patients with dose reduction, 125 to 100 mg (*N* = 311)	Patients without dose reduction (*N* = 550)^†^	Pooled ITT population (*N* = 875)
Age, years			
Mean (range)	59.9 (32–88)	60.0 (30–89)	59.9 (30–89)
< 65, *n* (%)	192 (61.7)	366 (66.5)	571 (65.3)
≥ 65, *n* (%)	119 (38.3)	184 (33.5)	304 (34.7)
Race, *n* (%)			
White	211 (67.8)	451 (82.0)	672 (76.8)
Black	7 (2.3)	14 (2.5)	21 (2.4)
Asian	77 (24.8)	64 (11.6)	145 (16.6)
Other^‡^	16 (5.1)	21 (3.8)	37 (4.2)
Weight, mean (range), kg	67.9 (33.0–123.0)	72.4 (39.7–156.8)	70.6 (33.0–156.8)
Height, mean (range), cm	159.7 (134.6–182.9)	161.3 (138.0–187.0)	160.7 (134.6–187.0)
Measurable disease, *n* (%)	225 (72.3)	435 (79.1)	671 (76.7)
Prior chemotherapy for advanced/metastatic disease, *n* (%)	32 (10.3)	68 (12.4)	113 (12.9)
Disease site, *n* (%)			
Visceral	146 (46.9)	299 (54.4)	452 (51.7)
Nonvisceral	165 (53.1)	251 (45.6)	423 (48.3)
Bone only	84 (27.0)	117 (21.3)	206 (23.5)
Number of disease sites involved, *n* (%)			
1	103 (33.1)	167 (30.4)	272 (31.1)
2	86 (27.7)	141 (25.6)	231 (26.4)
3	69 (22.2)	131 (23.8)	203 (23.2)
4	37 (11.9)	68 (12.4)	106 (12.1)
5	16 (5.1)	29 (5.3)	47 (5.4)
Disease stage at initial diagnosis,^ǁ^*n* (%)			
I	28 (9.0)	47 (8.5)	77 (8.8)
II	99 (31.8)	152 (27.6)	257 (29.4)
III	45 (14.5)	95 (17.3)	141 (16.1)
IV	75 (24.1)	148 (26.9)	224 (25.6)
Other	8 (2.6)	13 (2.4)	21 (2.4)
Unknown/missing	23 (7.4)	46 (8.4)	71 (8.1)
Not collected	33 (10.6)	49 (8.9)	84 (9.6)

*ITT* intent-to-treat

*Patients in PALOMA-1 and PALOMA-2 received palbociclib plus letrozole; patients in PALOMA-3 had endocrine-resistant metastatic breast cancer and received palbociclib plus fulvestrant

^†^Data from patients who started on a dose < 125 mg are not included in the table

^‡^Not reported/missing patients

^ǁ^Stage at initial diagnosis was not collected for PALOMA-1

Demographics and baseline characteristics were generally similar among patients who had a dose reduction compared with all patients in the pooled population who received palbociclib and with patients who did not require dose reduction; however, there were more Asian patients in the dose reduction cohort (24.8% vs 16.6%). Mean body weight was lower in the dose reduction group compared with the non–dose reduction group (67.9 and 72.4 kg, respectively). Median (range) baseline hemoglobin was similar in patients who did and did not require dose reduction (12.6 [8.3–15.5] and 13.0 [8.9–16.9] g/dL, respectively). Median (range) baseline absolute neutrophil count (ANC, × 10^9^/L) was 3.0 (1.3–78.1) and 3.9 (1.5–12.4), and median (range) platelet count (× 10^9^/L) was 230.0 (119.0–455.0) and 254.0 (90.0–632.0) in patients who did and did not require dose reduction, respectively.

Of the 311 palbociclib-treated patients who required a palbociclib dose reduction from 125 to 100 mg, 34 (10.9%) permanently discontinued due to treatment-emergent AEs. Hematologic AEs that led to permanent discontinuation were neutropenia (*n* = 11), anemia, and thrombocytopenia (both *n* = 1). No patient discontinued because of febrile neutropenia.

### Timeline to dose reduction

**Fig. 1:**
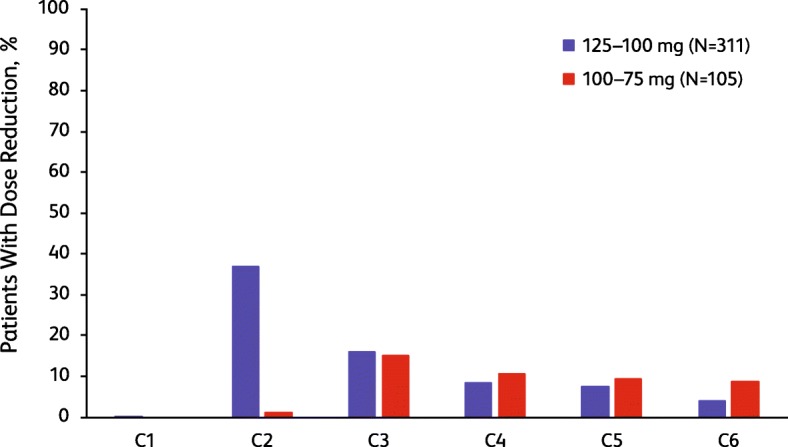
Timeline to first and second dose reduction by treatment cycle. Bars represent the percentage of patients who required dose reduction in each cycle. First dose reduction was from 125 to 100 mg. Second dose reduction was from 100 to 75 mg. C, cycle occurring after the dose reduction

The majority of dose reductions occurred early in palbociclib treatment (Fig. 1). Approximately 50% of dose reductions from 125 to 100 mg occurred by the third treatment cycle, and another 20% had occurred by C6. For patients who required a second dose reduction, from 100 to 75 mg, approximately half of these dose reductions had occurred by C6. Median time to first dose reduction of palbociclib in this pooled analysis was 70 days (range 15.0–1269.0 days). Median time to second dose reduction was 106.0 days (range 29.0–699.0 days).

### Adverse events after dose reduction of palbociclib from 125 to 100 mg

**Fig. 2:**
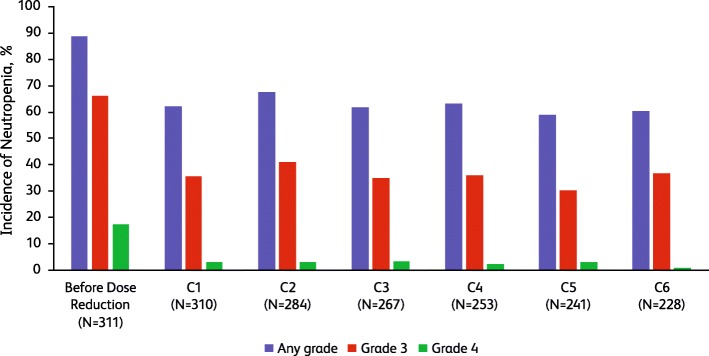
Incidence of neutropenia (neutropenia includes the following preferred terms: neutropenia and neutrophil count decreased) before and after palbociclib dose reduction from 125 to 100 mg. C, cycle occurring after the dose reduction

After palbociclib dose reduction from 125 to 100 mg, the frequency of neutropenia decreased during the first cycle following dose reduction, with the severity of neutropenia shifting to lower grades (Fig. 2). By the 6th cycle after the first dose reduction, the percentage of patients with grades 3 and 4 neutropenia decreased from 66.2% and 17.4% before dose reduction, respectively, to 36.8% and 0.9%.

The frequency and severity of leukopenia decreased during the first cycle following dose reduction and remained low (Table 2). Before dose reduction of palbociclib, the frequency of febrile neutropenia was 2.3%; only 1 patient had febrile neutropenia 5 cycles following dose reduction. The frequency of grade 3/4 thrombocytopenia and grade 3 anemia was 1.9% and 2.6%, respectively, before dose reduction; no grade 4 anemia was reported. After dose reduction, the frequency of all-grade and grade 3/4 thrombocytopenia decreased. The frequency of all-grade anemia was stable following dose reduction, but the frequency of grade 3 anemia decreased.

**Table 2:** Incidence of hematologic adverse events before/after palbociclib dose reduction from 125 to 100 mg

Adverse event	Before dose reduction (*N* = 311)			After dose reduction																	
				Cycle 1 (*N* = 310)			Cycle 2 (*N* = 284)			Cycle 3 (*N* = 267)			Cycle 4 (*N* = 253)			Cycle 5 (*N* = 241)			Cycle 6 (*N* = 228)		
	Grade, *n* (%)			Grade, *n* (%)			Grade, *n* (%)			Grade, *n* (%)			Grade, *n* (%)			Grade, *n* (%)			Grade, *n* (%)		
	All	3	4	All	3	4	All	3	4	All	3	4	All	3	4	All	3	4	All	3	4
Leukopenia*	130 (41.8)	86 (27.7)	2 (0.6)	88 (28.4)	25 (8.1)	0	83 (29.2)	22 (7.7)	0	71 (26.6)	22 (8.2)	0	64 (25.3)	17 (6.7)	0	60 (24.9)	12 (5.0)	0	56 (24.6)	15 (6.6)	0
Thrombocytopenia^†^	48 (15.4)	5 (1.6)	1 (0.3)	36 (11.6)	3 (1.0)	0	29 (10.2)	3 (1.1)	0	23 (8.6)	2 (0.7)	0	21 (8.3)	2 (0.8)	0	20 (8.3)	0	0	20 (8.8)	1 (0.4)	0
Anemia^‡^	38 (12.2)	8 (2.6)	0	55 (17.7)	5 (1.6)	0	54 (19.0)	3 (1.1)	0	49 (18.4)	2 (0.7)	0	43 (17.0)	2 (0.8)	0	38 (15.8)	2 (0.8)	0	39 (17.1)	1 (0.4)	0
Febrile neutropenia	7 (2.3)	6 (1.9)	1 (0.3)	0	0	0	0	0	0	0	0	0	0	0	0	1 (0.4)	1 (0.4)	0	0	0	0

*Leukopenia includes the following preferred terms: leukopenia or white blood cell count decreased

^†^Thrombocytopenia includes the following preferred terms: platelet count decreased or thrombocytopenia

^‡^Anemia includes the following preferred terms: anemia or hematocrit decreased or hemoglobin decreased

### Adverse events after dose reduction of palbociclib from 100 to 75 mg

**Fig. 3:**
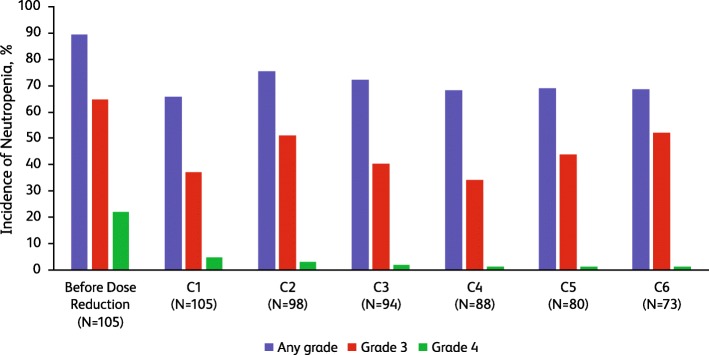
Incidence of neutropenia (neutropenia includes the following preferred terms: neutropenia and neutrophil count decreased) before and after palbociclib dose reduction from 100 to 75 mg. C, cycle occurring after the dose reduction

Among patients who had a dose reduction from 125 to 100 mg, 105/311 (33.8%) required a second palbociclib dose reduction from 100 to 75 mg. The rate of reported neutropenia AEs prior to dose reduction in this subgroup was 89.5% (94/105; all grades; Fig. 3). The frequency of neutropenia decreased during the first cycle following dose reduction, with a reduction in the severity of neutropenia. The rate of grade 4 neutropenia decreased from 21.9% before dose reduction to 1.4% within 6 cycles following dose reduction.

The frequency and severity of leukopenia decreased following dose reduction, and the frequency of grade 3 leukopenia remained low from C1 to C6 (Table 3); no grade 4 leukopenia was reported before or after the second dose reduction. The frequency of grade 3 thrombocytopenia and anemia was 2.9% and 3.8%, respectively, before dose reduction from 100 to 75 mg (no incidence of grade 4 for either AE). The frequency and severity of thrombocytopenia and anemia decreased after dose reduction; no grade 3 thrombocytopenia was reported after dose reduction, and there was a gradual decrease in the frequency of grade 3 anemia. One patient had grade 3 febrile neutropenia before the 2nd dose reduction, and 1 patient had grade 3 febrile neutropenia during the 5th cycle following dose reduction.

**Table 3:** Incidence of hematologic adverse events before/after palbociclib dose reduction from 100 to 75 mg

Adverse event	Before dose reduction (*N* = 105)			After dose reduction																	
				Cycle 1 (*N* = 105)			Cycle 2 (*N* = 98)			Cycle 3 (*N* = 94)			Cycle 4 (*N* = 88)			Cycle 5 (*N* = 80)			Cycle 6 (*N* = 73)		
	Grade, *n* (%)			Grade, *n* (%)			Grade, *n* (%)			Grade, *n* (%)			Grade, *n* (%)			Grade, *n* (%)			Grade, *n* (%)		
	All	3	4	All	3	4	All	3	4	All	3	4	All	3	4	All	3	4	All	3	4
Leukopenia*	36 (34.3)	23 (21.9)	0	29 (27.6)	12 (11.4)	0	26 (26.5)	8 (8.2)	0	22 (23.4)	6 (6.4)	0	21 (23.9)	6 (6.8)	0	16 (20.0)	5 (6.3)	0	17 (23.3)	6 (8.2)	0
Thrombocytopenia^†^	15 (14.3)	3 (2.9)	0	12 (11.4)	0	0	11 (11.2)	0	0	6 (6.4)	0	0	6 (6.8)	0	0	6 (7.5)	0	0	6 (8.2)	0	0
Anemia^‡^	11 (10.5)	4 (3.8)	0	13 (12.4)	3 (2.9)	0	11 (11.2)	2 (2.0)	0	11 (11.7)	1 (1.1)	0	9 (10.2)	0	0	7 (8.8)	0	0	5 (6.8)	1 (1.4)	0
Febrile neutropenia	1 (1.0)	1 (1.0)	0	0	0	0	0	0	0	0	0	0	0	0	0	1 (1.3)	1 (1.3)	0	0	0	0

*Leukopenia includes the following preferred terms: leukopenia or white blood cell count decreased

^†^Thrombocytopenia includes the following preferred terms: platelet count decreased or thrombocytopenia

^‡^Anemia includes the following preferred terms: anemia or hematocrit decreased or hemoglobin decreased

### Adverse events after dose reduction of palbociclib in Asian vs non-Asian patients, by body mass index, and by age

An analysis of Asian patients from the pooled analysis, defined as those patients with Asian ethnicity regardless of geographic region, showed that this subpopulation had a slightly higher frequency of hematologic AEs before the first dose reduction versus non-Asian patients (Supplementary Table 4). The frequency of neutropenia was 98.7% versus 85.5% in Asian versus non-Asian patients, respectively (leukopenia, 35.1% vs 44.0%; thrombocytopenia, 22.1% vs 13.2%; anemia, 13.0% vs 12.0%). Both subgroups had a decrease in the frequency and severity of hematologic AEs following dose reduction from 125 to 100 mg. Febrile neutropenia was reported in 1 Asian patient before dose reduction, and only 1 patient had febrile neutropenia (grade 3) during the fifth cycle after dose reduction. In non-Asian patients, the frequency of febrile neutropenia before dose reduction was 2.6%. There were no cases of febrile neutropenia following dose reduction.

An analysis by body mass index (BMI) showed that patients with BMI > 30 kg/m^2^ had a slightly lower frequency of hematologic AEs before the first dose reduction compared with those who had BMI > 25–30 kg/m^2^ or BMI ≤ 25 kg/m^2^ (Supplementary Table 5). Frequency of hematologic AEs in patients with BMI ≤ 25 kg/m^2^, BMI > 25–30 kg/m^2^, and BMI > 30 kg/m^2^ was as follows: neutropenia—90.5%, 90.7%, and 82.9%; leukopenia—43.8%, 42.3%, and 36.8%; thrombocytopenia—18.2%, 14.4%, and 11.8%; and anemia—14.6%, 13.4%, and 6.6%. All BMI subpopulations had a decrease in frequency and severity of hematologic AEs, including febrile neutropenia, following palbociclib dose reduction.

Adverse events before and after dose reduction were also analyzed in subgroups of patients aged < 65 and ≥ 65 years (Supplementary Table 6). In the month before dose reduction, the frequency of neutropenia, leukopenia, and thrombocytopenia in patients aged < 65 and ≥ 65 years was comparable: 90.6% and 85.7%, 43.2% and 39.5%, and 13.5% and 18.5%, respectively. The incidence of anemia was lower in patients aged < 65 compared with those ≥ 65 years: 9.4% and 16.8%, respectively, during this time period. Following dose reduction, the incidence of all-grade and grade 3/4 hematologic AEs, including febrile neutropenia, decreased in both age group cohorts.

## Discussion

In general, dose modifications successfully reduced hematologic AEs in patients who received palbociclib in addition to an aromatase inhibitor or fulvestrant. The primary reason for a first reduction in palbociclib dose was the occurrence of an AE. Dose reductions in response to hematologic AEs were made per protocol-specified guidelines (Supplementary Table 2); these data do not support the use of dose reduction for lower grades of hematologic AEs than those specified by the PALOMA clinical trials. Median time to a first dose reduction was 70 days. A decrease in the frequency and severity of hematologic AEs, including neutropenia, leukopenia, and thrombocytopenia, was observed following palbociclib dose reduction regardless of ethnicity, BMI, or age. The rate of permanent discontinuation due to neutropenia was low. The frequency of anemia was stable following dose reduction, with a gradual decrease in frequency observed for grade 3 anemia. Relatively few patients experienced febrile neutropenia before dose reduction, and this number decreased further following dose reduction. A decrease in AE frequency and severity was also observed following the second palbociclib dose reduction (100 to 75 mg). Moreover, there were no reported secondary hematologic malignancies. These findings support previous reports demonstrating that hematologic AEs can be successfully managed via palbociclib dose modification (without apparent loss of efficacy) and appropriate supportive care [10–12], and that growth factor support is not necessary. It is important to note that palbociclib should be started at a dose of 125 mg/day; our findings should not be interpreted as supportive of a lower initial dose.

CDK4/6 inhibitors impede cell cycle progression by interfering with the formation of CDK-cyclin complexes and thus block G1/S transition [13, 14]. Therefore, neutropenia resulting from CDK4/6 inhibitors is reversible, and when these inhibitors are held, cell cycle progression can recommence. This is in contrast to neutropenia induced by chemotherapy agents, which induce DNA damage and promote bone marrow apoptosis [14]. The observed rebound in neutrophil counts and reduction in incidence and severity of neutropenia observed following dose modification in this pooled, post hoc analysis is therefore reflective of the mechanism of action of palbociclib and likely partly explains the low incidence of neutropenic fever in these patients.

In the previous pooled analysis, there was no evidence of cumulative or delayed toxicities with palbociclib plus ET. Over a 3-year period, the rate of permanent discontinuations associated with palbociclib treatment was 8.3%, which suggests that the toxicities associated with palbociclib treatment can be well managed [9]. In the present analysis, the rate of permanent discontinuations due to AEs for palbociclib-treated patients who required a dose reduction from 125 to 100 mg was 10.9%, similar to that previously reported [9]. Both pooled analyses suggest that dose modifications reduced the incidence and severity of hematologic AEs, further supporting a manageable safety profile with palbociclib treatment.

A landmark analysis of PALOMA-2 showed that PFS was similar among patients who experienced dose reduction versus those who experienced no dose reduction in the palbociclib plus letrozole treatment arm of the trial [15]. In PALOMA-3, PFS was similar in patients receiving palbociclib plus fulvestrant regardless of whether they had ≥ 1 dose reduction or no dose reduction due to neutropenia [16]. Subgroup analyses of PALOMA-2 and PALOMA-3 also demonstrated that Asian and non-Asian patients had similar PFS outcomes, even though Asian patients had more dose reductions [17]. This finding is likely due to higher palbociclib exposure in Asian versus non-Asian patients because of lower palbociclib clearance in Asian patients [10, 17]. Dose modification due to AEs does not appear to negatively impact the efficacy of palbociclib plus ET in patients with HR+/HER2− ABC, but does reduce the severity and incidence of AEs. The findings of this pooled analysis, together with the efficacy data outlined above, further support the use of palbociclib dose modification to manage AEs.

The effect of palbociclib dose reduction on the frequency and severity of hematologic AEs regardless of ethnicity is supported by a previous analysis of PALOMA-2, in which higher rates of hematologic AEs were observed in Asian versus non-Asian patients but were effectively managed with dose adjustments [17]. Higher rates of neutropenia may be due to a lower baseline ANC. An analysis of PALOMA-2 showed that patients with a lower baseline ANC were more likely to experience grade 3/4 neutropenia when treated with palbociclib plus letrozole [15]. Furthermore, Asian patients in PALOMA-2 and PALOMA-3 had baseline ANC that were 18% and 20% lower, respectively, compared with non-Asian patients [17, 18]. The higher incidence and severity of hematologic AEs, including neutropenia, in Asian patients may be interrelated with the higher incidence of AEs in patients with a lower baseline BMI (≤ 25 kg/m^2^). In PALOMA-2, mean body weight was lower among Asian versus non-Asian patients [17]. Furthermore, in PALOMA-2 and PALOMA-3, median body weight and BMI were lower among Japanese patients compared with the overall population [12].

Aside from Asian ethnicity and a lower baseline ANC, no other predictive markers for toxicity were identified in the current analysis. Patients with lower BMI had a slightly higher frequency of neutropenia than patients with higher BMI. Age < 65 and ≥ 65 years appeared to have little impact on the rates of hematologic AEs. In a previous report, patients ≥ 75 years of age had a higher frequency of neutropenia compared with those < 75 years of age [19]. However, the present study did not analyze AEs in this patient subgroup due to the small sample size. Of note, some risk factors may confound each other, and further study to understand combinations of risk factors associated with an increased risk of grade 3/4 neutropenia leading to dose reduction may be informative for clinicians. In a recent study of patients treated with palbociclib who had received prior chemotherapy, 100 mg palbociclib was associated with lower rates of grade 3/4 neutropenia than 125 mg; further, correlation of response by dose suggested that use of biomarkers such as pRB and Ki67 could indicate the use of palbociclib treatment and reduce toxicity [20]. Additional study of the use of biomarkers to aid in the management of AEs is therefore warranted.

Treatment with palbociclib plus letrozole maintained health-related quality of life (QoL) in PALOMA-2 [21]. Although neutropenia is the most frequent AE associated with palbociclib treatment, no statistical difference in change from baseline in Functional Assessment of Cancer Therapy–Breast score was found in patients with or without neutropenia [21]. Moreover, QoL can be maintained through dose modifications to manage AEs [4]. The relatively low rate of permanent discontinuations due to AEs observed in the present analysis, compared with the overall rate in real-world practice, supports the findings of these previous studies and the use of dose modifications to manage AEs.

This analysis is subject to limitations, including its post hoc nature. Because of the stringent inclusion and exclusion criteria, the generalizability of these findings to the clinical setting may be limited. Neutropenia management in the PALOMA trials included dose reduction and/or interruption; due to the inherent complexities in analyzing dose interruptions, this manuscript only analyzed dose reductions. The results could therefore be confounded by dose interruption. Furthermore, in the clinic, patients with breast cancer often present with comorbidities and are receiving concomitant medications, which may influence the generalizability of these findings to real-world practice.

## Conclusions

In summary, the incidence and severity of hematologic AEs associated with palbociclib treatment can be managed through dose reductions, irrespective of ethnicity, BMI, or age, in patients with HR+/HER2− ABC. Of note, these dose reductions do not appear to influence the efficacy of palbociclib. Early recognition and management of AEs are critical for their successful management and to allow continued treatment and optimization of treatment outcomes.

## Supplementary information

**Additional file 1 **: **Table S1.** Summary of the PALOMA Clinical Trial Data. **Table S2.** Schedule of Per Protocol Palbociclib Dose Reductions and Modifications. **Table S3.** Time Points for Assessing Frequency of AEs Both Before and After Palbociclib Dose Reductions. **Table S4.** Incidence of Hematologic Adverse Events Before/After Palbociclib Dose Reduction From 125 to 100 mg: Asian/non-Asian. **Table S5.** Incidence of Hematologic Adverse Events Before/After Palbociclib Dose Reduction From 125 to 100 mg: BMI. **Table S6.** Incidence of Hematologic Adverse Events Before/After Palbociclib Dose Reduction From 125 to 100 mg: Age. **Figure S1.** Study Design of PALOMA-1, PALOMA-2, and PALOMA-3.

## Data Availability

Upon request, and subject to certain criteria, conditions, and exceptions (see https://www.pfizer.com/science/clinical-trials/trial-data-and-results for more information), Pfizer will provide access to individual de-identified participant data from Pfizer-sponsored global interventional clinical studies conducted for medicines, vaccines, and medical devices (1) for indications that have been approved in the USA and/or EU or (2) in programs that have been terminated (i.e., development for all indications has been discontinued). Pfizer will also consider requests for the protocol, data dictionary, and statistical analysis plan. Data may be requested from Pfizer trials 24 months after study completion. The de-identified participant data will be made available to researchers whose proposals meet the research criteria and other conditions, and for which an exception does not apply, via a secure portal. To gain access, data requestors must enter into a data access agreement with Pfizer.

## Abbreviations

ABC: Advanced breast cancer (includes metastatic breast cancer)
AE: Adverse event
ANC: Absolute neutrophil count
BMI: Body mass index
C: Cycle
CDK4/6: Cyclin-dependent kinase 4/6
ER+: Estrogen receptor–positive
ET: Endocrine therapy
HER2−: Human epidermal growth factor receptor 2–negative
HR+: Hormone receptor–positive
PFS: Progression-free survival
QoL: Quality of life
