# Supplementary material for: Hematologic adverse events following palbociclib dose reduction in patients with hormone receptor–positive/human epidermal growth factor receptor 2–negative advanced breast cancer: pooled analysis from randomized phase 2 and 3 studies
Source: Breast Cancer Res. 2020 Mar 12;22:27. doi: 10.1186/s13058-020-01263-0 (PMC7068918; doi:10.1186/s13058-020-01263-0)
Supplement: Supplementary file 1 — Additional file 1 : Table S1. Summary of the PALOMA Clinical Trial Data. Table S2. Schedule of Per Protocol Palbociclib Dose Reductions and Modifications. Table S3. Time Points for Assessing Frequency of AEs Both Before and After Palbociclib Dose Reductions. Table S4. Incidence of Hematologic Adverse Events Before/After Palbociclib Dose Reduction From 125 to 100 mg: Asian/non-Asian. Table S5. Incidence of Hematologic Adverse Events Before/After Palbociclib Dose Reduction From 125 to 100 mg: BMI. Table S6. Incidence of Hematologic Adverse Events Before/After Palbociclib Dose Reduction From 125 to 100 mg: Age. Figure S1. Study Design of PALOMA-1, PALOMA-2, and PALOMA-3. [file 13058_2020_1263_MOESM1_ESM.pdf]

Supplementary Table 1. Summary of the PALOMA Clinical Trial Data.

	PALOMA-1* N=165		PALOMA-2† N=666		PALOMA-3‡ N=521	
**Patient population**	First-line ER+/HER2– ABC		First-line ER+/HER2– ABC		Second- or later-line HR+/HER2– ABC	
**Treatment arms**	**PAL+LET n=84**	**LET n=81**	**PAL+LET n=444**	**PBO+LET n=222**	**PAL+FUL n=347**	**PBO+FUL n=174**
**Median PFS, mo**	20.2	10.2	27.6	14.5	11.2	4.6
**Hazard ratio**	0.488		0.563		0.50	
**95% CI**	0.319–0.748		0.461–0.687		0.40–0.62	
***P* value**	*P*=0.0004		*P*<0.000001		*P*<0.0001	
**Most common AE in the PAL arm**	Neutropenia		Neutropenia		Neutropenia	
**Median palbociclib dose intensity (range), %‖**	100 (95–100)	NA	93.0 (40–110)	99.6 (56–105)	89.8 (22–107)	100 (80–100)

ABC=advanced breast cancer; AE=adverse event; CI=confidence interval; ER+=estrogen receptor–positive; FUL=fulvestrant; HER2–=human epidermal growth factor receptor 2–negative; HR+=hormone receptor–positive; LET=letrozole; NA=not available; PAL=palbociclib; PBO=placebo; PFS=progression-free survival.
*From Finn et al. *Lancet Oncol.* 2015;16:25-35.
†From Rugo et al. *Breast Cancer Res Treat.* 2019;174(3):719-729.
‡From Turner et al. *N Engl J Med.* 2018;379:1926-1936.
‖From Diéras et al. *J Natl Cancer Inst.* 2019;11(4):419-430.

Supplementary Table 2. Schedule of Per Protocol Palbociclib Dose Reductions and Modifications.

	PALOMA-1<br>N=165	PALOMA-2<br>N=666	PALOMA-3<br>N=521
Uncomplicated grade 3 neutropenia (ANC <1000/mm^3)	---	No dose adjustments	Dose reduction 1 dose level if neutrophil recovery is delayed beyond 7 days*,†
Grade 3 neutropenia (ANC <1000/mm^3) associated with a documented infection or fever ≥38.5°C	Dose reduction 1 dose level	Dose reduction 1 dose level	Dose reduction 1 dose level; dose reduction 2 dose levels‡ if neutrophil recovery is delayed beyond 7 days*
Grade 4 neutropenia (ANC <500/mm^3)	Dose reduction 1 dose level	Dose reduction 1 dose level	Dose reduction 1 dose level; dose reduction 2 dose levels‡ in case of recurrent grade 4 event*
Grade 3 or 4‖ thrombocytopenia (platelet count <50,000/mm^3)	Dose reduction 1 dose level	Dose reduction 1 dose level	Dose reduction 1 dose level; dose reduction 2 dose levels‡ in case of recurrent grade ≥3 event
Grade ≥3 non-hematologic toxicity (including, nausea, vomiting, diarrhea, and hypertension only if persisting despite optimal medical treatment)	Dose reduction 1 dose level	Dose reduction 1 dose level	Dose reduction 1 dose level; dose reduction 2 dose levels‡ if repeated toxicity is seen in the next cycle or if recovery from grade 3 is delayed beyond 7 days*
Grade 3 QTc prolongation (QTc ≥501 msec on at least 2 separate ECGs)	---	Dose interruption until QTc <501 msec; resume at same dose level if reversible cause identified and	Dose interruption until QTc <501 msec; resume at same dose level if reversible cause identified and

		at next lower dose if no reversible cause identified	at next lower dose if no reversible cause identified

ANC=absolute neutrophil count; ECG=electrocardiogram.

*If recovery of neutrophils to ≥1000/mm^3 or platelet count to ≥50,000/mm^3 takes longer than 2 weeks (which may include dose holding due to toxicity, the scheduled week off treatment and up to 7 days of cycle delay), then reduce by 2 dose levels.

†If uncomplicated grade 3 neutropenia recurs in 2 consecutive cycles, after recovery as per retreatment criteria (ANC ≥1000/mm^3 and no fever), treatment may restart at the next lower dose level at investigator's discretion.

‡If no further dose reduction is possible (ie, patient is already receiving 75 mg/d according to schedule 3/1) consider changing the schedule to 75 mg/d 2 weeks on/2 weeks off, or discontinue palbociclib/placebo and continue with fulvestrant alone.

‖Dose reduction occurred with grade 4 thrombocytopenia (platelet count <25,000/mm^3) for PALOMA-1 and PALOMA-2.

Supplementary Table 3. Time Points for Assessing Frequency of AEs Both Before and After Palbociclib Dose Reductions.

	AE Assessment Period
**Before Dose Reduction**	AEs reported within 30 days before reduction
**After Dose Reduction**	
Cycle 1	AEs reported within 28 days after dose reduction
Cycle 2	AEs reported 29–56 days after dose reduction
Cycle 3	AEs reported 57–84 days after dose reduction
Cycle 4	AEs reported 85–112 days after dose reduction
Cycle 5	AEs reported 113–140 days after dose reduction
Cycle 6	AEs reported 141–168 days after dose reduction

AE=adverse event.

Supplementary Table 4. Incidence of Hematologic Adverse Events Before/After Palbociclib Dose Reduction From 125 to 100 mg: Asian/non-Asian.

	Before Dose Reduction			After Dose Reduction																	
Asian	Before (N=77) Grade, n (%)			Cycle 1 (N=77) Grade, n (%)			Cycle 2 (N=68) Grade, n (%)			Cycle 3 (N=62) Grade, n (%)			Cycle 4 (N=58) Grade, n (%)			Cycle 5 (N=56) Grade, n (%)			Cycle 6 (N=52) Grade, n (%)		
Adverse Event	All	3	4	All	3	4	All	3	4	All	3	4	All	3	4	All	3	4	All	3	4
Neutropenia*	76 (98.7)	52 (67.5)	21 (27.3)	50 (64.9)	35 (45.5)	4 (5.2)	46 (67.6)	34 (50.0)	3 (4.4)	38 (61.3)	24 (38.7)	4 (6.5)	35 (60.3)	20 (34.5)	4 (6.9)	34 (60.7)	19 (33.9)	1 (1.8)	34 (65.4)	21 (40.4)	1 (1.9)
Leukopenia†	27 (35.1)	20 (26.0)	0	21 (27.3)	7 (9.1)	0	16 (23.5)	4 (5.9)	0	13 (21.0)	5 (8.1)	0	11 (19.0)	3 (5.2)	0	11 (19.6)	3 (5.4)	0	14 (26.9)	7 (13.5)	0
Thrombocytopenia‡	17 (22.1)	3 (3.9)	0	9 (11.7)	1 (1.3)	0	9 (13.2)	1 (1.5)	0	6 (9.7)	0	0	6 (10.3)	0	0	7 (12.5)	0	0	7 (13.5)	1 (1.9)	0
Anemia§	10 (13.0)	2 (2.6)	0	15 (19.5)	2 (2.6)	0	14 (20.6)	1 (1.5)	0	12 (19.4)	1 (1.6)	0	11 (19.0)	1 (1.7)	0	10 (17.9)	1 (1.8)	0	10 (19.2)	1 (1.9)	0
Febrile neutropenia	1 (1.3)	1 (1.3)	0	0	0	0	0	0	0	0	0	0	0	0	0	1 (1.8)	1 (1.8)	0	0	0	0
Non-Asian	Before (N=234) Grade, n (%)			Cycle 1 (N=233) Grade, n (%)			Cycle 2 (N=216) Grade, n (%)			Cycle 3 (N=205) Grade, n (%)			Cycle 4 (N=195) Grade, n (%)			Cycle 5 (N=185) Grade, n (%)			Cycle 6 (N=176) Grade, n (%)		
Adverse Event	All	3	4	All	3	4	All	3	4	All	3	4	All	3	4	All	3	4	All	3	4
Neutropenia*	200 (85.5)	154 (65.8)	33 (14.1)	143 (61.4)	75 (32.2)	5 (2.1)	146 (67.6)	83 (38.4)	6 (2.8)	127 (62.0)	69 (33.7)	5 (2.4)	125 (64.1)	71 (36.4)	2 (1.0)	108 (58.4)	54 (29.2)	6 (3.2)	104 (59.1)	63 (35.8)	1 (0.6)
Leukopenia†	103 (44.0)	66 (28.2)	2 (0.9)	67 (28.8)	18 (7.7)	0	67 (31.0)	18 (8.3)	0	58 (28.3)	17 (8.3)	0	53 (27.2)	14 (7.2)	0	49 (26.5)	9 (4.9)	0	42 (23.9)	8 (4.5)	0
Thrombocytopenia‡	31 (13.2)	2 (0.9)	1 (0.4)	27 (11.6)	2 (0.9)	0	20 (9.3)	2 (0.9)	0	17 (8.3)	2 (1.0)	0	15 (7.7)	2 (1.0)	0	13 (7.0)	0	0	13 (7.4)	0	0
Anemia§	28 (12.0)	6 (2.6)	0	40 (17.2)	3 (1.3)	0	40 (18.5)	2 (0.9)	0	37 (18.0)	1 (0.5)	0	32 (16.4)	1 (0.5)	0	28 (15.1)	1 (0.5)	0	29 (16.5)	0	0
Febrile neutropenia	6 (2.6)	5 (2.1)	1 (0.4)	0	0	0	0	0	0	0	0	0	0	0	0	0	0	0	0	0	0

*Neutropenia includes the following Preferred Terms: Neutropenia or Neutrophil count decreased.
†Leukopenia includes the following Preferred Terms: Leukopenia or White blood cell count decreased.
‡Thrombocytopenia includes the following Preferred Terms: Platelet count decreased or Thrombocytopenia.
§Anemia includes the following Preferred Terms: Anemia or Hematocrit decreased or Hemoglobin decreased.

Supplementary Table 5. Incidence of Hematologic Adverse Events Before/After Palbociclib Dose Reduction From 125 to 100 mg: BMI.

	Before Dose Reduction			After Dose Reduction																	
**BMI ≤25 kg/m²**	**Before (N=137) Grade, n (%)**			**Cycle 1 (N=136) Grade, n (%)**			**Cycle 2 (N=121) Grade, n (%)**			**Cycle 3 (N=113) Grade, n (%)**			**Cycle 4 (N=106) Grade, n (%)**			**Cycle 5 (N=101) Grade, n (%)**			**Cycle 6 (N=94) Grade, n (%)**		
**Adverse Event**	**All**	**3**	**4**	**All**	**3**	**4**	**All**	**3**	**4**	**All**	**3**	**4**	**All**	**3**	**4**	**All**	**3**	**4**	**All**	**3**	**4**
**Neutropenia***	124 (90.5)	89 (65.0)	30 (21.9)	90 (66.2)	53 (39.0)	6 (4.4)	84 (69.4)	51 (42.1)	6 (5.0)	69 (61.1)	41 (36.3)	2 (1.8)	68 (64.2)	35 (33.0)	5 (4.7)	55 (54.5)	24 (23.8)	4 (4.0)	61 (64.9)	40 (42.6)	2 (2.1)
**Leukopenia†**	60 (43.8)	41 (29.9)	0	37 (27.2)	8 (5.9)	0	33 (27.3)	9 (7.4)	0	30 (26.5)	9 (8.0)	0	27 (25.5)	5 (4.7)	0	20 (19.8)	2 (2.0)	0	25 (26.6)	8 (8.5)	0
**Thrombocytopenia‡**	25 (18.2)	3 (2.2)	0	18 (13.2)	2 (1.5)	0	16 (13.2)	2 (1.7)	0	12 (10.6)	1 (0.9)	0	11 (10.4)	0	0	10 (9.9)	0	0	11 (11.7)	1 (1.1)	0
**Anemia§**	20 (14.6)	4 (2.9)	0	26 (19.1)	2 (1.5)	0	26 (21.5)	3 (2.5)	0	24 (21.2)	2 (1.8)	0	20 (18.9)	2 (1.9)	0	18 (17.8)	2 (2.0)	0	18 (19.1)	1 (1.1)	0
**Febrile neutropenia**	4 (2.9)	4 (2.9)	0	0	0	0	0	0	0	0	0	0	0	0	0	1 (1.0)	1 (1.0)	0	0	0	0
**BMI >25–30 kg/m²**	**Before (N=97) Grade, n (%)**			**Cycle 1 (N=97) Grade, n (%)**			**Cycle 2 (N=88) Grade, n (%)**			**Cycle 3 (N=84) Grade, n (%)**			**Cycle 4 (N=80) Grade, n (%)**			**Cycle 5 (N=76) Grade, n (%)**			**Cycle 6 (N=74) Grade, n (%)**		
**Adverse Event**	**All**	**3**	**4**	**All**	**3**	**4**	**All**	**3**	**4**	**All**	**3**	**4**	**All**	**3**	**4**	**All**	**3**	**4**	**All**	**3**	**4**
**Neutropenia***	88 (90.7)	67 (69.1)	13 (13.4)	58 (59.8)	32 (33.0)	2 (2.1)	63 (71.6)	43 (48.9)	1 (1.1)	55 (65.5)	30 (35.7)	6 (7.1)	54 (67.5)	34 (42.5)	1 (1.3)	56 (73.7)	32 (42.1)	3 (3.9)	48 (64.9)	27 (36.5)	0
**Leukopenia†**	41 (42.3)	28 (28.9)	1 (1.0)	32 (33.0)	10 (10.3)	0	33 (37.5)	11 (12.5)	0	27 (32.1)	10 (11.9)	0	24 (30.0)	7 (8.8)	0	27 (35.5)	7 (9.2)	0	21 (28.4)	4 (5.4)	0
**Thrombocytopenia‡**	14 (14.4)	1 (1.0)	0	10 (10.3)	0	0	9 (10.2)	0	0	9 (10.7)	0	0	7 (8.8)	0	0	8 (10.5)	0	0	8 (10.8)	0	0
**Anemia§**	13 (13.4)	3 (3.1)	0	17 (17.5)	0	0	18 (20.5)	0	0	15 (17.9)	0	0	14 (17.5)	0	0	12 (15.8)	0	0	14 (18.9)	0	0
**Febrile neutropenia**	2 (2.1)	2 (2.1)	0	1 (1.0)	0	0	0	0	0	0	0	0	0	0	0	0	0	0	0	0	0
**BMI >30 kg/m²**	**Before (N=76) Grade, n (%)**			**Cycle 1 (N=76) Grade, n (%)**			**Cycle 2 (N=75) Grade, n (%)**			**Cycle 3 (N=70) Grade, n (%)**			**Cycle 4 (N=67) Grade, n (%)**			**Cycle 5 (N=64) Grade, n (%)**			**Cycle 6 (N=60) Grade, n (%)**		

Adverse Event	All	3	4	All	3	4	All	3	4	All	3	4	All	3	4	All	3	4	All	3	4
**Neutropenia***	63 (82.9)	49 (64.5)	11 (14.5)	44 (57.9)	25 (32.9)	1 (1.3)	45 (60.0)	23 (30.7)	2 (2.7)	41 (58.6)	22 (31.4)	1 (1.4)	38 (56.7)	22 (32.8)	0	31 (48.4)	17 (26.6)	0	29 (48.3)	17 (28.3)	0
**Leukopenia†**	28 (36.8)	16 (21.1)	1 (1.3)	19 (25.0)	7 (9.2)	0	17 (22.7)	2 (2.7)	0	14 (20.0)	3 (4.3)	0	13 (19.4)	5 (7.5)	0	13 (20.3)	3 (4.7)	0	10 (16.7)	3 (5.0)	0
**Thrombocytopenia‡**	9 (11.8)	1 (1.3)	1 (1.3)	8 (10.5)	1 (1.3)	0	4 (5.3)	1 (1.3)	0	2 (2.9)	1 (1.4)	0	3 (4.5)	2 (3.0)	0	2 (3.1)	0	0	1 (1.7)	0	0
**Anemia§**	5 (6.6)	1 (1.3)	0	12 (15.8)	3 (3.9)	0	10 (13.3)	0	0	10 (14.3)	0	0	9 (13.4)	0	0	8 (12.5)	0	0	7 (11.7)	0	0
**Febrile neutropenia**	1 (1.3)	0	1 (1.3)	0	0	0	0	0	0	0	0	0	0	0	0	0	0	0	0	0	0

BMI=body mass index.
*Neutropenia includes the following Preferred Terms: Neutropenia or Neutrophil count decreased.
†Leukopenia includes the following Preferred Terms: Leukopenia or White blood cell count decreased.
‡Thrombocytopenia includes the following Preferred Terms: Platelet count decreased or Thrombocytopenia.
§Anemia includes the following Preferred Terms: Anemia or Hematocrit decreased or Hemoglobin decreased.

Supplementary Table 6. Incidence of Hematologic Adverse Events Before/After Palbociclib Dose Reduction From 125 to 100 mg: Age.

	Before Dose Reduction			After Dose Reduction																	
**Age <65 Years**	**Before (N=192) Grade, n (%)**			**Cycle 1 (N=192) Grade, n (%)**			**Cycle 2 (N=179) Grade, n (%)**			**Cycle 3 (N=169) Grade, n (%)**			**Cycle 4 (N=158) Grade, n (%)**			**Cycle 5 (N=147) Grade, n (%)**			**Cycle 6 (N=138) Grade, n (%)**		
**Adverse Event**	**All**	**3**	**4**	**All**	**3**	**4**	**All**	**3**	**4**	**All**	**3**	**4**	**All**	**3**	**4**	**All**	**3**	**4**	**All**	**3**	**4**
**Neutropenia***	174 (90.6)	134 (69.8)	35 (18.2)	124 (64.6)	65 (33.9)	6 (3.1)	129 (72.1)	75 (41.9)	7 (3.9)	112 (66.3)	62 (36.7)	7 (4.1)	100 (63.3)	55 (34.8)	5 (3.2)	84 (57.1)	39 (26.5)	4 (2.7)	79 (57.2)	43 (31.2)	1 (0.7)
**Leukopenia†**	83 (43.2)	55 (28.6)	2 (1.0)	58 (30.2)	15 (7.8)	0	57 (31.8)	14 (7.8)	0	51 (30.2)	14 (8.3)	0	44 (27.8)	9 (5.7)	0	41 (27.9)	7 (4.8)	0	37 (26.8)	8 (5.8)	0
**Thrombocytopenia‡**	26 (13.5)	2 (1.0)	1 (0.5)	21 (10.9)	1 (0.5)	0	17 (9.5)	1 (0.6)	0	15 (8.9)	1 (0.6)	0	14 (8.9)	1 (0.6)	0	13 (8.8)	0	0	12 (8.7)	1 (0.7)	0
**Anemia§**	18 (9.4)	5 (2.6)	0	31 (16.1)	3 (1.6)	0	31 (17.3)	3 (1.7)	0	29 (17.2)	2 (1.2)	0	24 (15.2)	1 (0.6)	0	19 (12.9)	1 (0.7)	0	21 (15.2)	1 (0.7)	0
**Febrile neutropenia**	4 (2.1)	3 (1.6)	1 (0.5)	0	0	0	0	0	0	0	0	0	0	0	0	1 (0.7)	1 (0.7)	0	0	0	0
**Age ≥65 Years**	**Before (N=119) Grade, n (%)**			**Cycle 1 (N=118) Grade, n (%)**			**Cycle 2 (N=105) Grade, n (%)**			**Cycle 3 (N=98) Grade, n (%)**			**Cycle 4 (N=95) Grade, n (%)**			**Cycle 5 (N=94) Grade, n (%)**			**Cycle 6 (N=90) Grade, n (%)**		
**Adverse Event**	**All**	**3**	**4**	**All**	**3**	**4**	**All**	**3**	**4**	**All**	**3**	**4**	**All**	**3**	**4**	**All**	**3**	**4**	**All**	**3**	**4**
**Neutropenia***	102 (85.7)	72 (60.5)	19 (16.0)	69 (58.5)	45 (38.1)	3 (2.5)	63 (60.0)	42 (40.0)	2 (1.9)	53 (54.1)	31 (31.6)	2 (2.0)	60 (63.2)	36 (37.9)	1 (1.1)	58 (61.7)	34 (36.2)	3 (3.2)	59 (65.6)	41 (45.6)	1 (1.1)
**Leukopenia†**	47 (39.5)	31 (26.1)	0	30 (25.4)	10 (8.5)	0	26 (24.8)	8 (7.6)	0	20 (20.4)	8 (8.2)	0	20 (21.1)	8 (8.4)	0	19 (20.2)	5 (5.3)	0	19 (21.1)	7 (7.8)	0
**Thrombocytopenia‡**	22 (18.5)	3 (2.5)	0	15 (12.7)	2 (1.7)	0	12 (11.4)	2 (1.9)	0	8 (8.2)	1 (1.0)	0	7 (7.4)	1 (1.1)	0	7 (7.4)	0	0	8 (8.9)	0	0
**Anemia§**	20 (16.8)	3 (2.5)	0	24 (20.3)	2 (1.7)	0	23 (21.9)	0	0	20 (20.4)	0	0	19 (20.0)	1 (1.1)	0	19 (20.2)	1 (1.1)	0	18 (20.0)	0	0
**Febrile neutropenia**	3 (2.5)	3 (2.5)	0	0	0	0	0	0	0	0	0	0	0	0	0	0	0	0	0	0	0

*Neutropenia includes the following Preferred Terms: Neutropenia or Neutrophil count decreased.

†Leukopenia includes the following Preferred Terms: Leukopenia or White blood cell count decreased.

‡Thrombocytopenia includes the following Preferred Terms: Platelet count decreased or Thrombocytopenia.

§Anemia includes the following Preferred Terms: Anemia or Hematocrit decreased or Hemoglobin decreased.

Supplementary Figure 1. Study Design of PALOMA-1, PALOMA-2, and PALOMA-3.

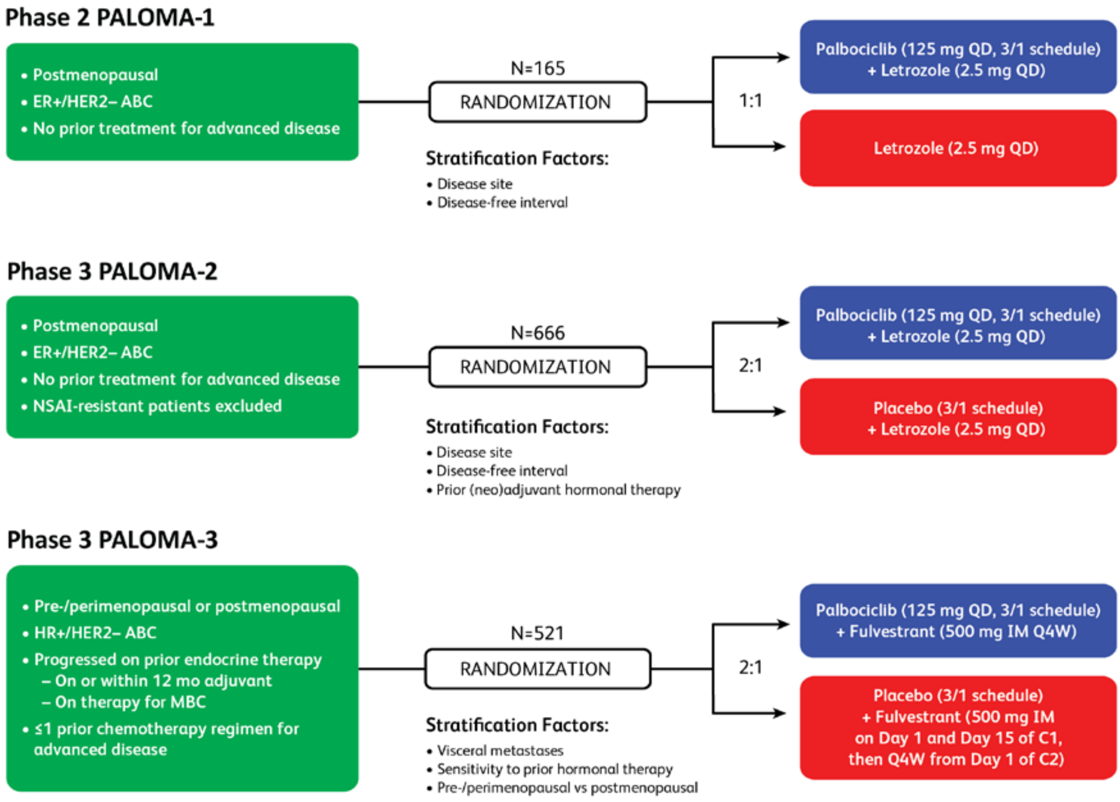

ABC=advanced breast cancer; C=cycle; D=day; ER+=estrogen receptor–positive; HER2–=human epidermal growth factor receptor 2–negative; HR+=hormone receptor–positive; IM=intramuscular; MBC=metastatic breast cancer; NSAI=nonsteroidal aromatase inhibitor; QD=once daily; Q4W=every 4 weeks.
